# A de novo heterozygous variant in ACOX1 gene cause Mitchell syndrome: the first case in China and literature review

**DOI:** 10.1186/s12920-023-01577-w

**Published:** 2023-07-03

**Authors:** Mengxiao Shen, Qian Chen, Yanyan Gao, Hongyu Yan, Shuo Feng, Xinna Ji, Xue Zhang

**Affiliations:** 1 Department of Neurology, Affiliated Children’s Hospital of Capital Institute of Pediatrics, Beijing, China; 2 Chinese Academy of Medical Sciences & Peking Union Medical College, Beijing, China; 3 State Key Laboratory of Medical Molecular Biology, Institute of Basic Medical Sciences, Chinese Academy of Medical Sciences & Peking Union Medical College, Beijing, China

**Keywords:** Mitchell syndrome (MITCH), ACOX1 gene, Recurrent rash, Gait instability, Hearing loss, Autonomic symptoms, Very-long-chain fatty acid (VLCFA), Gain of function (GOF)

## Abstract

**Background:**

Mitchell syndrome (MITCH) is a rare autosomal dominant hereditary disorder, characterized by episodic demyelination, sensorimotor polyneuropathy and hearing loss. MITCH is caused by heterozygous mutation in the ACOX1 gene, which encodes straight-chain acyl-CoA oxidase, on chromosome 17q25.1.  Only 5 unrelated patients have been reported so far, and no reports from China. Here, we describe the first MITCH case in a Chinese individual.

**Case presentation:**

A 7-year-old girl initially presented with diffuse desquamatory rash at age 3. Her clinical symptoms in order of presentation were diffuse desquamatory rash, gait instability, ptosis with photophobia, hearing loss, abdominal pain, diarrhea, nausea, and dysuria. Genetic analysis demonstrated that the patient carried a heterozygous variant c.710A>G(p.Asp237Ser) in the ACOX1 gene, which can cause MITCH symptoms. This is the first MITCH case with gastrointestinal and urinary tract symptoms. After administrating N acetylcysteine amide (NACA), some symptoms were relieved and the patient’s condition improved.

**Conclusion:**

This is the first MITCH case in the Chinese population, and we expanded the genotype spectrum of it. The p.Asp237Ser may be a mutational hotspot in ACOX1 regardless of race. In terms of diagnosis, patients with recurrent rash, gait instability, and hearing loss with some autonomic symptoms should raise the suspicion of MITCH and proper and prompt treatment should be given.

**Supplementary Information:**

The online version contains supplementary material available at 10.1186/s12920-023-01577-w.

## Background

Mitchell syndrome (MITCH, OMIM #618,960) is a rare and progressive autosomal dominant hereditary disease characterized by episodic demyelination, sensorimotor polyneuropathy, and hearing loss. In 2020, Chung et al. first demonstrated that MITCH is caused by the de novo heterozygous missense mutation in the acyl-CoA oxidase (ACOX1) on chromosome 17q25.1 [1].

The ACOX1 gene encodes peroxisomal acyl-CoA oxidase, the first and rate-limiting enzyme of the very-long-chain fatty acid (VLCFA) β-oxidation pathway in peroxisomes. Hydrogen peroxide is a byproduct of acyl-CoA oxidase activity [2]. Homozygous mutation in the ACOX1 gene is loss-of-function and causes peroxisome ACOX1 deficiency (OMIM#264,470), which is characterized by rapid and severe neurological damage as evidenced by hypotonia and seizures, enhanced reflexes, increased muscle tone, severe and recurrent seizures, progressive blindness and deafness, and VLCFA accumulation due to disturbances in fatty acid metabolism.

Most children with ACOX1 deficiency do not survive past early childhood [3, 4]. However, the heterozygous mutations in the ACOX1 gene in MITCH patients increase reactive oxygen species (ROS) levels but do not alter VLCFA. Studies in flies carrying a heterozygous mutation in ACOX1 presented elevated protein dimerization and appeared to be resistant to protein turnover [1]. The mutant protein also increased enzyme activity compared to the wildtype one, elucidating a gain-of-function (GOF) effect [1].

Up to now, only 5 unrelated patients with MITCH have been described in North American [1, 5, 6] but no reports from China. Here, we describe a MITCH patient with a de novo heterozygous variant in ACOX1 gene, which verifies GOF effect of the pathogenic variant (c.710 A > G, p.Asp237 Ser). To our knowledge, this is the first report of MITCH in China.

## Case presentation

A 7-year-old girl with diffuse desquamatory rash, gait instability, ptosis with photophobia, hearing loss, abdominal pain, diarrhea, nausea, and dysuria was admitted to our institution. She was the firstborn of nonconsanguineous parents with unremarkable medical and family histories. Her younger sister is healthy. Pregnancy was uneventful, and she was born via cesarean section due to oligohydramnios at 39 weeks gestational age. Birth weight was 3950 g, length 50 cm, and head 33 cm. APGAR score was 9,10 at 1st, 5th minutes, respectively.

At 3 years of age, she initially presented with a raised, nonpruritic rash around the eye and mouth that quickly spread to the upper limb and eventually developed desquamation and pruritic. There was an antecedent infection. Staphylococcal scalded skin syndrome (SSSS) was diagnosed at a local hospital and recovered after hospitalization. At 3 years and 4 months, she had a repeat episode of diffused rash followed by cerebellar ataxia gait and then inability to stand or even sit up without assistance. During this episode, she developed positive neurological signs, including mild proximal lower extremity weakness, lower limb areflexia, and failure to complete heel-knee-shin and finger-nose tests. Total spine and brain magnetic resonance imaging (MRI), as well as electroencephalogram (EEG), were unremarkable. Cerebrospinal fluid (CSF) analysis was unremarkable. Electromyography (EMG) illustrated myogenic damage. On the second episode, she was diagnosed with pediatric acute cerebellar ataxia and dermatomyositis at a local hospital and treated with initial empiric intravenous dexamethasone 7.5 mg for 3 days with no obvious response, and was transferred to our hospital. Extensive investigations to evaluate disorders were conducted, including autoimmune(total lymphocyte count, lymphocyte and immunoglobulins subsets, lupus anticoagulant, double-stranded DNA antibodies, antinuclear antibodies, complement C3 and C4, rheumatoid factor, demyelinating neuropathy autoantibody panels, sensory and motor neuropathy, autoimmune encephalitis autoantibody panel on blood and CSF, etc.), mitochondrial function and other metabolic factors (serum amino acids, lactate, pyruvate, urine organic acids, methylmalonic acid, targeted sequencing of nuclear and mitochondrial genes cause mitochondrial disturbances, etc.), infectious and inflammatory factors, toxic exposure, thyroid-stimulating hormone, and thyroid hormone. None of the above were abnormal except for lymphocyte subsets. Abdominal ultrasound, whole-body CT scan, and 18 F-fluorodeoxyglucose PET precluded a neoplastic/paraneoplastic origin. The EMG was normal, moreover, mildly elevated muscle enzymes and normal muscle biopsy excluded myogenic origin. We considered recurrent autoimmune cerebellar ataxia and dermatomyositis and a 3 days course of 20 mg/kg of intravenous Methylprednisolone was attempted. Fortunately, she had a robust response to treatment and was discharged on oral Prednisone (tapering from 25 mg/d) plus Pidotimod.

The patient had approximately 2 years of stable situation after discharge, with remission of rash and gait difficulties. Nevertheless, after drug withdrawal for 3 months, her rash and disability to walk relapsed due to a prodromal infection. She was admitted to a local institution for reevaluation of diagnosis and therapeutic management. During the third episode, she developed a new symptom of ptosis with photosensitivity. Visual field testing was unremarkable, but visual evoked potential was abnormal. Combined with previous history, she was suspected of ichthyosis polyneuritis ataxia syndrome. There was no obvious response and deterioration due to influenza, despite symptomatic treatment. What’s more, 2 months later she developed new symptoms of bilateral hearing loss, ultimately needed hearing aids placement, and then was transferred to various hospitals for about a year only to be repeatedly misdiagnosed and treated ineffectively.

**Fig. 1:**
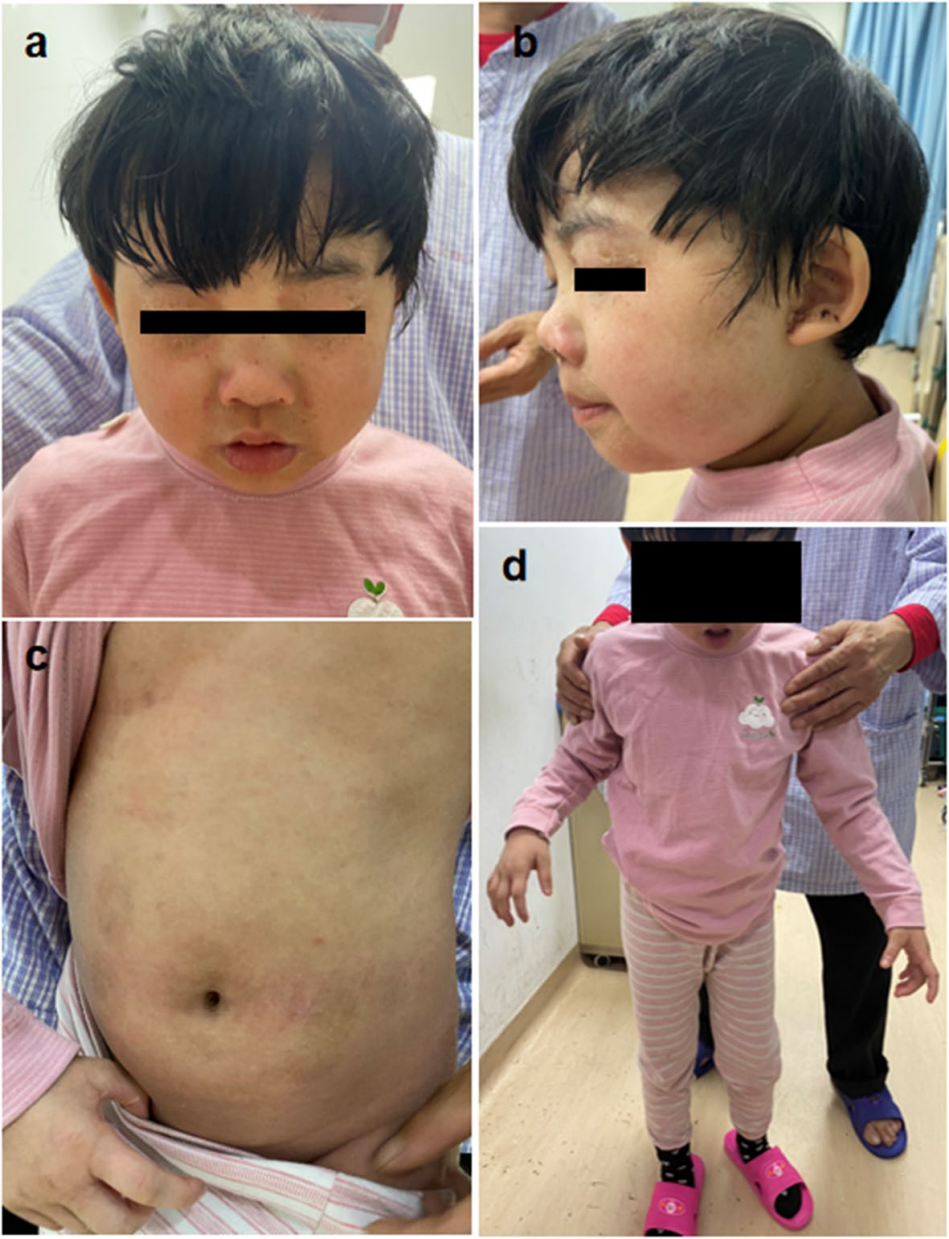
Patient’s photographs: **A-C** diffuse desquamatory rash **D** gait instability

**Fig. 2:**
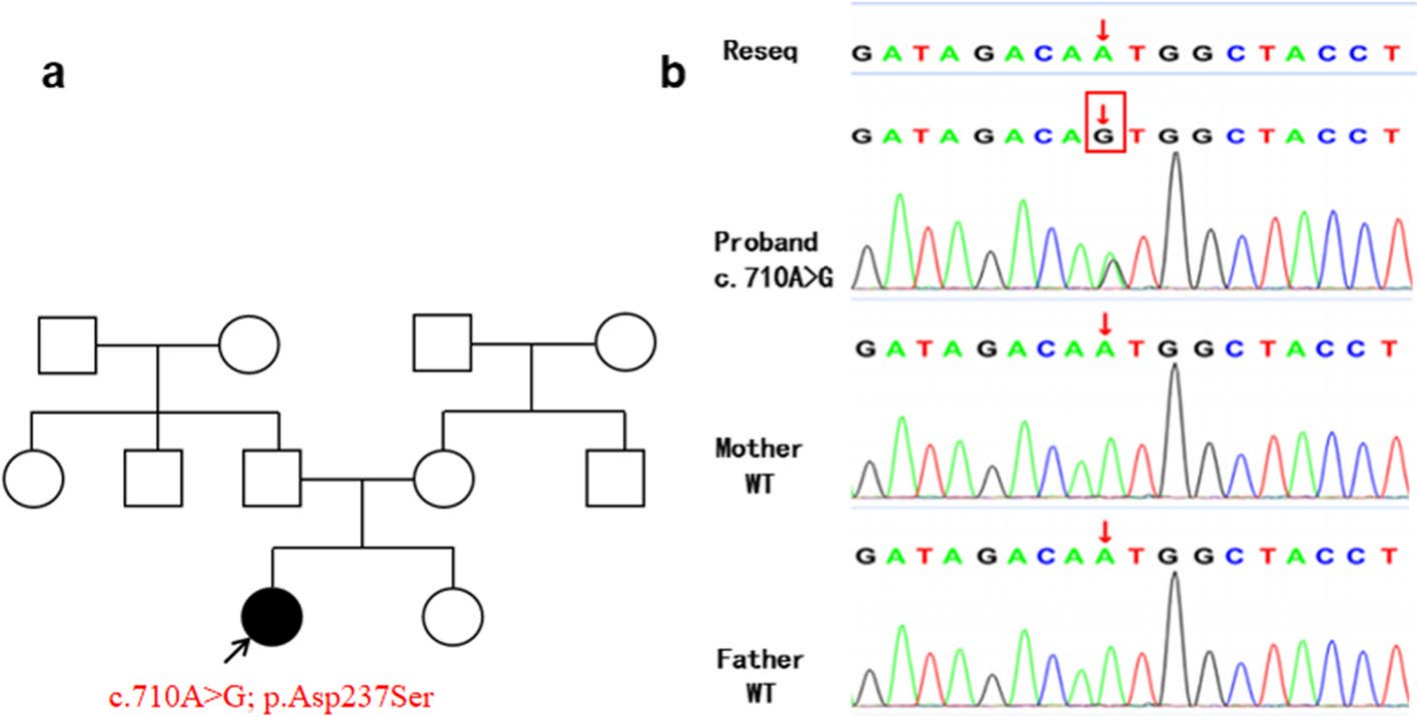
Clinical trios whole-exome sequencing chromatogram: Red box indicates the heterozygous missense mutation in ACOX1 gene in the proband (c.710 A > G, p.Asp237Ser

Ten days before admission, she had a precipitous deterioration in rash (Fig. 1A-C) and ability to walk (Fig. 1D), as well as a choking cough after drinking. Seven days before admission, she developed autonomic symptoms including abdominal pain and diarrhea, nausea, and dysuria and then was transferred to our institution again. The total lymphocyte count and lymphocyte subsets decreased, bilateral auditory brainstem evoked potential threshold increased and latency prolonged, sensory and motor conduction velocity slowed and sensory nerve action potential amplitudes decreased as well as EMG suggested peripheral nerve damage. Given waxed and waned courses and progressive symptoms, we conducted whole exome sequencing (WES) to identify the etiology. WES was carried out on genomic DNA extracted from blood, a deep next generation sequencing (NGS) with an average reading depth of 30×, IDT’s xGen Exome Research Panel v2 was selected and identified a pathogenic de novo variant in ACOX1 in the proband (c.710 A > G, p.Asp237Ser) (Fig. 2). We also performed the detection of base repeat regions in 10 causative genes of ataxia without abnormal result. Considered that ACOX1 was known to involve only in an autosomal recessive peroxisomal ACOX1 deficiency, VLCFA were tested and the concentration was normal, indicating no loss of peroxisomal proteins and no decrease in enzyme activity (Table S1). Thus, the heterozygous de novo missense mutation has a new pathomechanism different from previously reported peroxisomal ACOX1 deficiency.

Supported by previous good response to immunotherapy and the abnormal total lymphocyte count and lymphocyte subsets, Methylprednisolone (a 3 days course of 20 mg/kg) was re-administered and oral Prednisone (50 mg/d) supplementation subsequently, which showed a good response except for gait difficulties. Then, oral N acetylcysteine amide (NACA) supplementation (3 g, twice daily) was initiated to attenuate levels of ROS, with a good response. At this point, she had a good recovery and was discharged after 14 days of admission. After 11 months of follow-up, her condition improved gradually, particularly in terms of rash, gastrointestinal, urinary, and gait difficulties, and she was able to have a partially autonomous life, attend school and partially maintain social relationships.

## Discussion and conclusions

In this study, we describe the first MITCH case in China presented with desquamatory rash, gait instability, photophobia, hearing loss, abdominal pain and diarrhea, diarrhea, nausea and dysuria symptoms and a good response to immunotherapy and antioxidant. It is noteworthy that gastrointestinal and urinary tract symptoms in MITCH cases have never been proposed.

MITCH is a progressive disorder characterized by episodic demyelination, sensorimotor polyneuropathy and hearing loss, caused by heterozygous variant in ACOX1(c.710 A > G, p.Asp237Ser) that encodes straight-chain acyl-CoA oxidase, on chromosome 17q25.1. Studies in flies carrying ACOX1 heterozygous variant presented elevated protein dimerization and seemed to be resistant to protein turnover [1]. The mutant protein also increased enzyme activity compared to the wildtype one, demonstrating a GOF effect that produces increased levels of ROS in glia and brings about axonal loss [1], which is different from peroxisomal ACOX1 deficiency caused by homozygous mutation in ACOX1 [3, 7]. The normal level of VLCFA in our case also verified the activity of variant protein was not decreased, which is consistent with MITCH.

Up to now, only 5 unrelated patients found by trios WES have been reported, all of them displayed remarkably similar phenotypes, including progressive demyelination with sensorineural hearing loss, a waxing and waning courses, and were treated intermittently with immunomodulation and with NACA ultimately [1, 5, 6]. In addition to the above phenotypes, our case also suffered from gastrointestinal and urinary tract symptoms. Autonomic peripheral neuropathy caused by axonal loss implicates commonly in the gastrointestinal, urogenital and cardiovascular systems [8]. Of note, one of the previously reported patients developed acute onset of urinary retention, one of the manifestations of cauda equina syndrome, as her spinal MRI showed enhancement of cauda equina nerve roots [6], which was different from our case. Moreover, although total spine and brain MRI imaging in our case was normal so far, regularly monitoring the changes in MRI is indispensable in the future. The main clinical features of the 5 patients and our case are summarized in Table 1.

**Table 1:** Clinical features for our proband and 5 reported patients with GOF mutations in ACOX1

	Our proband	Patient 1	Patient 2	Patient 3	Patient 4	Patient 5
*ACOX1 variant*	de novo heterozygous missense variant (p.N237S)	de novo heterozygous missense variant (p.N237S)	de novo heterozygous missense variant (p.N237S)	de novo heterozygous missense variant (p.N237S)	de novo heterozygous missense variant (p.N237S)	de novo heterozygous missense variant (p.N237S)
*Disease onset*	3y	12y	9y	3y	14y	9y
*Disease course*	Waxing and waning	Waxing and waning then progressive	Waxing and waning then progressive	Waxing and waning then progressive	Waxing and waning	Waxing and waning
*Sensorimotor* *polyneuropathy*	Yes	Yes	Yes	Yes	Yes	Yes
*Hearing loss*	Yes	Yes	Yes	Yes	Yes	Yes
*Ocular Symptoms*	Yes	Yes	No	Yes	Yes	Yes
*Ataxia*	Yes	Yes	Yes	Yes	Yes	Yes
*Rash*	Yes	No	Yes	Yes	Yes	Yes
*Abdominal pain and diarrhea*	Yes	No	No	No	No	No
*Nausea*	Yes	No	No	No	No	No
*Dysuria*	Yes	No	No	No	Yes	No
*Cognition*	Normal	Normal→decreased	Normal→decreased	Decreased	Normal	Normal→decreased
*VLCFA* ^a^	Normal	Normal	Normal	Normal	Normal	Not reported
*White matter demyelination*	Normal	Normal→abnormal	Normal→abnormal	Normal→abnormal	Abnormal	Abnormal

^a^See Table S1 for specific values

The diagnosis of MITCH is challenging for it involves in multiple systems.

Our patient had been misdiagnosed as staphylococcal scalded skin syndrome and dermatomyositis, and the latter is similar to MITCH with respect to proximal extremity weakness, fluctuating courses and good response to immunotherapy. In terms of gait instability, we should differentiate it from acute cerebellar ataxia. Chung et al. indicated that the patient 2 had been misdiagnosed as autoimmune transverse myelitis with peripheral neuropathy and the patient 3 as Guillain-Barre Syndrome [1]. Furthermore, it is essential to distinguish it from disorders involving the skin and nervous system such as ichthyosis polyneuritis ataxia syndrome. All in all, patients manifested with recurrent rash, gait instability and peripheral neuropathy symptoms, waxing and waning courses as well as good response to immunotherapy should raise the suspicion of MITCH. Genetic testing allows definite diagnosis [9], and the p.Asp237Ser maybe mutational hotspot in ACOX1 regardless of race according to genotypes of all reported cases and our patient.

Early antioxidation and immune modulation were considered to be effective. Intravenous immunoglobulin have been shown to act as antioxidant and neuroprotective effects in vitro and in vivo [5]. Jafarpour et al. reported that mycophenolate mofetil could benefit patients with MITCH because of their antioxidant properties at low doses [5]. In addition, therapies targeting antioxidant effects such as carnitine and vitamin supplementation could be beneficial in patients with MITCH [5]. Chung et al. demonstrated that NACA, a potent antioxidant, able to penetrate the blood-brain-barrier, can strongly reverse the GOF of ACOX1, attenuate oxidative stress and suppress the production of ROS [1]. ROS are essential for immune cells in both physiological and pathogenic conditions [10], involved in the maturation, activation and differentiation of B-cell by regulating the signaling molecules in various molecular pathways [11], and also broadly associated with T-cell activation, hyporesponsiveness and apoptosis [12, 13]. It is obvious that the source, the kinetics, and the localization of ROS production all affect cell immune responses [14]. Therefore, deviations from the normal oxidative stress pathways appeared to bring about immune dysfunction so that immunomodulation therapies are effective in MITCH. In our case, both total lymphocyte count and lymphocyte subsets were decreased, indicating that ROS led to abnormalities in the immune system. Additionally, there were prodromal infections in the first and third episodes and her condition deteriorated with flus, which may be due to the immune system being provoked by infection. As consequence, chronic immune modulation is indispensable.

In conclusion, we describe the first Chinese MITCH case with a pathogenic de novo heterozygous variant in ACOX1, corroborating the GOF effect of ACOX1 (c.710 A > G, p.Asp237Ser). This is the first MITCH case presented with gastrointestinal symptoms and dysuria, expanding the mutation spectrum of MITCH. This study oftered us new insights into ACOX1-associated MITCH in the Chinese population. The p.Asp237Ser may be a mutational hotspot in ACOX1 regardless of race. Recurrent rash, gait instability, hearing loss with some autonomic symptoms should raise the suspicion of MITCH and prompt WES. Early antioxidation and immune modulation were regarded as effective. The prognosis has not been testified and our case was still under follow-up for long-term effect of immune therapy and antioxidant. Further studies are required to assess the long-term effect of medication and possible pathogenic mechanism.

## Supplementary Information

**Additional file 1: Table S1.** Normal VLCFA levels are observed in our proband.

## Data Availability

The datasets generated during the current study are deposited in NCBI Sequence Read Archive (SRA). The accession number is SRR21676605.

## Abbreviations

MITCH: Mitchell syndrome
WES: whole exome sequencing
ROS: reactive oxygen species
GOF: gain of function
VLCFA: very-long-chain fatty acid
NACA: N acetylcysteine amide
