# Supplementary material for: A de novo heterozygous variant in ACOX1 gene cause Mitchell syndrome: the first case in China and literature review
Source: BMC Med Genomics. 2023 Jul 3;16:156. doi: 10.1186/s12920-023-01577-w (PMC10318832; doi:10.1186/s12920-023-01577-w)
Supplement: Supplementary file 1 — Additional file 1: Table S1. Normal VLCFA levels are observed in our proband. [file 12920_2023_1577_MOESM1_ESM.docx]

**Table S1. Normal VLCFA levels are observed in our proband (Supplement, related to table 1)**

| **Plasma VLCFA** | **Proband(nmol/mL)** | **Medical reference range** |
| --- | --- | --- |
| C22:0 | 45.2 | ≤96.3 |
| C24:0 | 36.5 | ≤91.4 |
| C26:0 | 0.41 | ≤1.30 |
| C24/C22 | 0.81 | ≤1.39 |
| C26/C22 | 0.009 | ≤0.023 |
